# Supplementary material for: Septins associate with AP-3 to support trafficking to the vacuole/lysosome in yeast
Source: bioRxiv. 2026 Feb 14:2026.02.13.705769. Preprint. [Version 1] doi: 10.64898/2026.02.13.705769 (PMC12919031; doi:10.64898/2026.02.13.705769)
Supplement: Supplement 2 [file NIHPP2026.02.13.705769v1-supplement-2.pdf]

bioRxiv preprint doi: https://doi.org/10.64898/2026.02.13.705769; this version posted February 14, 2026. The copyright holder for this preprint (which was not certified by peer review) is the author/funder, who has granted bioRxiv a license to display the preprint in perpetuity. It is made available under aCC-BY 4.0 International license.

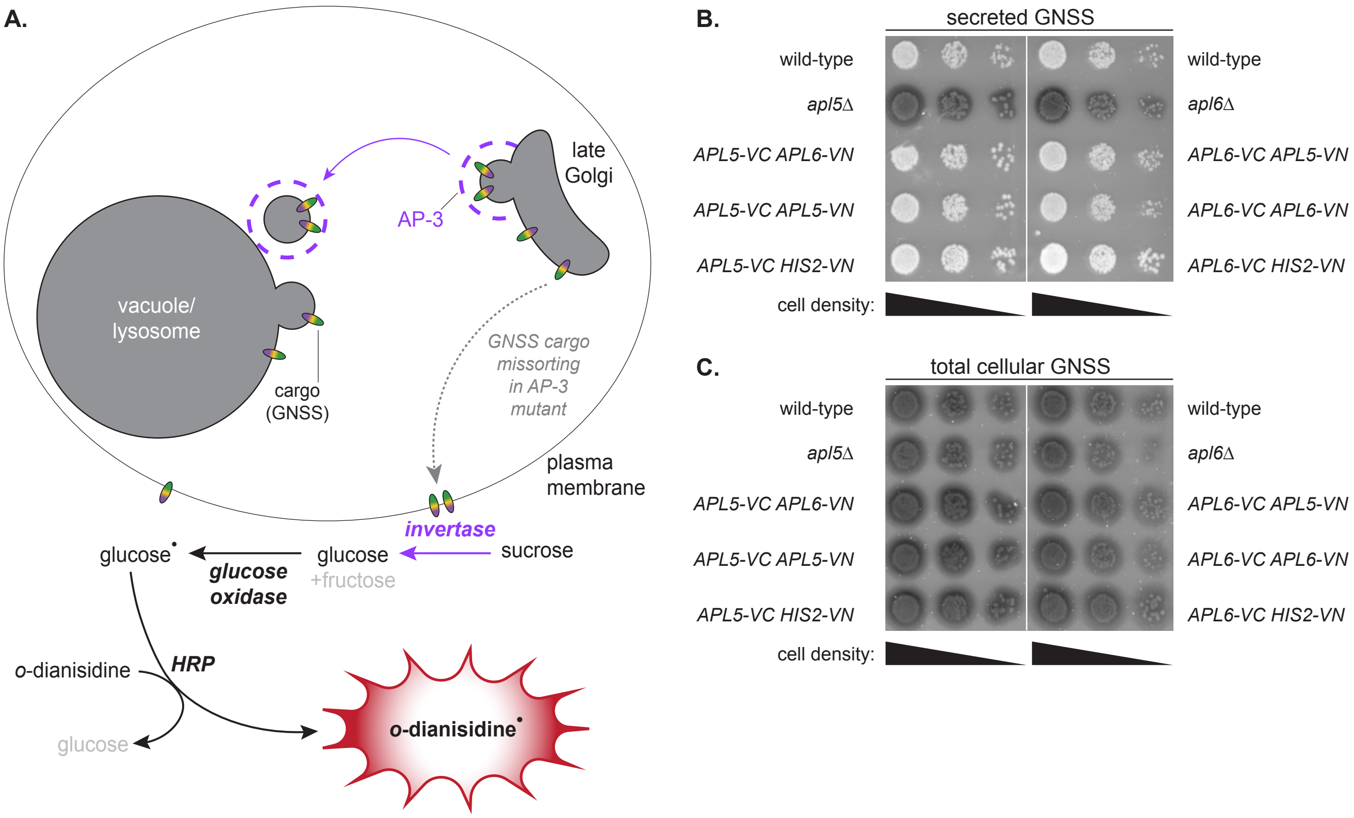

Supplemental Figure S1. BiFC fusions preserve AP-3 function

To assess whether BiFC fusions compromised AP-3 function, we examined trafficking of GNSS, a synthetic AP-3 cargo protein reporter that contains two invertase domains fused to its exoplasmic domain (Plemel et al., 2021; Leih et al., 2024). (A) Schematic diagram explaining the chromogenic invertase activity assay principle. GNSS normally traffics from the Golgi to the vacuole membrane via the AP-3 pathway. When AP-3 function is compromised, GNSS mislocalizes to the plasma membrane, where cell-surface invertase activity can be detected chromogenically using an overlay assay (Darsow et al., 2000). (B) Chromogenic overlay assay results for all BiFC strains used in Figure 1, showing normal GNSS internalization (lack of dark coloration) comparable to wild-type control; *apl5*Δ and *apl6Δ* serve as positive controls showing GNSS mislocalization (dark coloration). Representative images from n=3 independent experiments demonstrate that BiFC fusions—including both heterotypic pairings (Apl5-VN/Apl6-VC and reciprocal Apl6-VN/Apl5-VC) and homotypic pairings (Apl5-VN/Apl5-VC and Apl6-VN/Apl6-VC)—do not impair AP-3-dependent cargo sorting. The preservation of normal GNSS trafficking in cells with homotypic BiFC combinations demonstrates that irreversible Venus reconstitution does not trap AP-3 complexes in non-functional aggregates; rather, AP-3 retains the dynamic properties necessary for productive cargo sorting even after BiFC capture. (C) Chromogenic assay of the same cells shown in (B) after cellular lysis upon exposure to chloroform vapor, confirming equivalent GNSS expression across all strains.

bioRxiv preprint doi: https://doi.org/10.64898/2026.02.13.705769; this version posted February 14, 2026. The copyright holder for this preprint (which was not certified by peer review) is the author/funder, who has granted bioRxiv a license to display the preprint in perpetuity. It is made available under aCC-BY 4.0 International license.

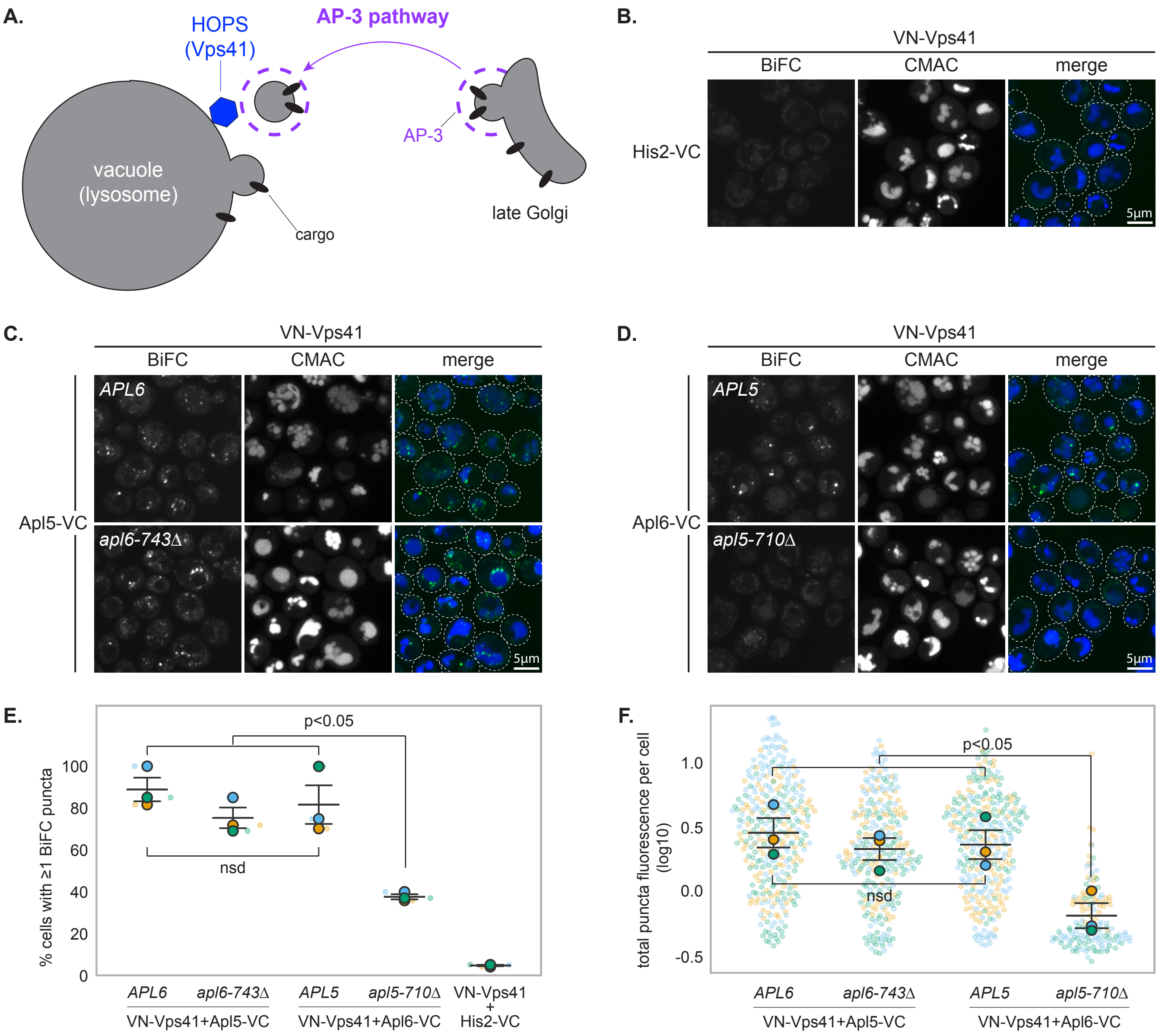

Supplemental Figure S2. The Apl5 δ subunit IDR promotes Vps41 engagement with AP-3

(A) Schematic showing HOPS complex at the vacuole and its established direct interaction with AP-3. Vps41 of the HOPS complex binds directly to the Apl5 IDR (Angers and Merz, 2009; Schoppe et al., 2020). (B-D) Representative confocal microscopy images of BiFC between VN-Vps41 (expressed from centromeric plasmid) and the indicated genomically-integrated VC fusions in wild-type or IDR deletion (*apl5-710Δ* or *apl6-743Δ*) backgrounds. His2-VC (B) serves as negative control. VN-Vps41 produced no signal with His2-VC but robust BiFC puncta with both Apl5-VC and Apl6-VC in wild-type cells, confirming the known Vps41-Apl5 interaction. While Vps41-Apl5 BiFC was expected based on direct binding, the Vps41-Apl6 signal likely reflects indirect proximity mediated through AP-3 spatial organization (Figure 1) rather than direct binding. Dashed ovals indicate cellular outlines. Scale bars, 5 µm. (E) Percentage of cells containing at least one BiFC punctum (≥50 cells scored per replicate; quantification as in Figure 1E). The Apl6 IDR deletion (*apl6-743Δ*) did not affect VN-Vps41/Apl5-VC association, whereas Apl5 IDR deletion (*apl5-710Δ*) reduced VN-Vps41/Apl6-VC association (puncta-positive cells: 81% to 37%, $p<0.05$), demonstrating that the Apl5 δ subunit IDR serves as the primary organizer of the AP-3-HOPS interface. AP-3 spatial organization potentially amplifies this interaction by bringing Apl6 into proximity with Vps41-bound Apl5. (F) Total puncta fluorescence per puncta-positive cell (quantification as in Figure 1F).

bioRxiv preprint doi: https://doi.org/10.64898/2026.02.13.705769; this version posted February 14, 2026. The copyright holder for this preprint (which was not certified by peer review) is the author/funder, who has granted bioRxiv a license to display the preprint in perpetuity. It is made available under aCC-BY 4.0 International license.

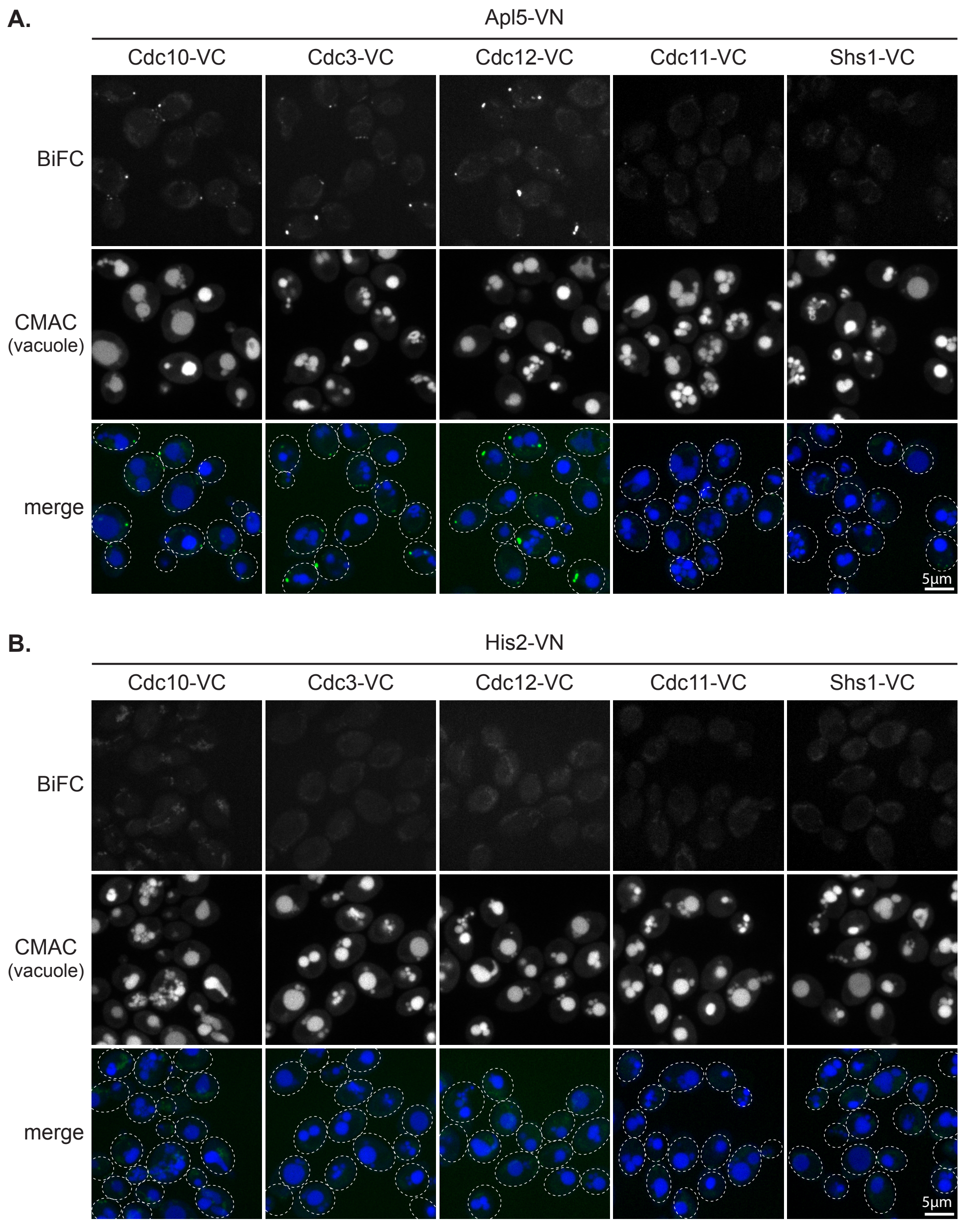

Supplemental Figure S3. AP-3 shows hierarchical BiFC proximity to septin subunits
(A) Representative confocal microscopy images of Apl5-VN paired with septin-VC fusions (Cdc10-VC, Cdc3-VC, Cdc12-VC, Cdc11-VC, Shs1-VC). Robust puncta are observed with core septins (Cdc10, Cdc3, Cdc12) while terminal septins (Cdc11, Shs1) show diminished signals, mirroring the hierarchy observed with Apl6-VN in Figure 3B. The differential BiFC pattern reveals that AP-3 shows preferential proximity to core septin subunits that form the central architectural scaffold of septin octamers. Core subunits Cdc10, Cdc3, and Cdc12 form the palindromic center of octamers, whereas Cdc11 and Shs1 occupy terminal positions (see Figure 3A). The consistent hierarchy observed with both Apl5-VN and Apl6-VN pairings (Figure 3B) supports the conclusion that AP-3 engages assembled septin octamers with preferential access to core architectural elements rather than terminal caps. Dashed ovals indicate cellular outlines in the merged images. Scale bars, 5 µm. (B) Negative controls showing no BiFC signal above background when septin-VC fusions are paired with His2-VN, confirming specificity of AP-3-septin proximity signals. Dashed ovals indicate cellular outlines in the merged images. Scale bars, 5 µm. Representative images from n=3 independent experiments.

bioRxiv preprint doi: https://doi.org/10.64898/2026.02.13.705769; this version posted February 14, 2026. The copyright holder for this preprint (which was not certified by peer review) is the author/funder, who has granted bioRxiv a license to display the preprint in perpetuity. It is made available under aCC-BY 4.0 International license.

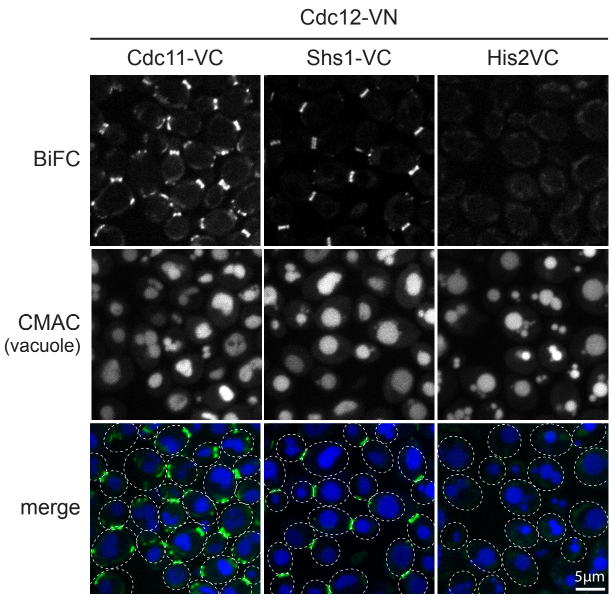

Supplemental Figure S4. Terminal septin VC fusions are properly expressed and functional
Representative confocal microscopy images showing robust BiFC signal between Cdc12-VN and either Cdc11-VC or Shs1-VC. Because Cdc11 and Shs1 are direct neighbors of Cdc12 within septin octamers (see Figure 3A; Bertin et al., 2008; Garcia et al., 2011; Weems and McMurray, 2017), this BiFC signal confirms that Cdc11-VC and Shs1-VC fusion proteins are properly expressed, correctly folded, and capable of assembling into septin octamers. The terminal septin VC fusions are also capable of participating in productive BiFC reconstitution when their interaction partners are appropriately positioned within ~7 nm. The robust BiFC observed in these positive control pairings demonstrates that the reduced BiFC signals observed between AP-3 subunits and terminal septins Cdc11-VC or Shs1-VC (Figure 3B-D) reflect genuine differences in spatial proximity or accessibility rather than technical artifacts such as impaired fusion protein expression, misfolding, or inability to reconstitute Venus fluorescence. This control is critical for interpreting the hierarchical interaction pattern, as it establishes that terminal septins are competent for BiFC but simply do not come into close proximity with AP-3 subunits as frequently as core septins do. Dashed ovals inidicate cellular outlines in megred images. Scale bars, 5 µm. Representative images from n=3 independent experiments.
